# Efficacy and safety of different PD-1 inhibitors in combination with lenvatinib in the treatment of unresectable primary liver cancer: a multicentre retrospective study

**DOI:** 10.1007/s12672-023-00708-0

**Published:** 2023-06-19

**Authors:** Qi-mei Li, Qing-can Sun, Yan Jian, Jing-zhe He, Hong-bo Zhu, Chang Hong, Lin Zeng, Rui-ning Li, Jia-ren Wang, Yan Li, Li-ya Chen, Xie Weng, Li Liu, Han-zhi Dong, Lu-shan Xiao, Hao Cui

**Affiliations:** 1 Department of Infectious Diseases, Nanfang Hospital, Southern Medical University, Guangzhou, 510515 China; 2 Department of Medical Oncology, The First Affiliated Hospital of Nanchang University, Nanchang, 330029 China; 3 Department of Oncology, The First Affiliated Hospital of University of South China, Hengyang, 421001 China; 4 Department of Medical Quality Management, Nanfang Hospital, Southern Medical University, Guangzhou, 510515 China; 5 Integrated Hospital of Traditional Chinese Medicine, Cancer Center, Southern Medicine University, Guangzhou, 510315 China; 6 Nanfang Hospital, Big Data Center, Southern Medical University, Guangzhou, 510515 China

**Keywords:** Primary liver cancer, Immune checkpoint inhibitors, Anti-angiogenesis drug, Combination therapy

## Abstract

Immune checkpoint inhibitors (ICIs) are safe and efficacious treatments for advanced primary liver cancer (PLC). The efficacy of different ICIs in the treatment of liver cancer remains unclear. The purpose of this study was to explore whether there is a difference in the efficacy and safety of various programmed cell death protein 1 (PD-1) inhibitors in combination with lenvatinib in the treatment of unresectable PLC. Patients with PLC treated with lenvatinib in combination with PD-1 inhibitors (camrelizumab, tislelizumab, sintilimab, or pembrolizumab) between January 2018 and December 2021 were retrospectively enrolled. Tumor response, adverse events, and grades were evaluated. Kaplan–Meier analysis and log-rank test were used to compare the overall survival and progression-free survival of patients treated with different PD-1 inhibitors. Cox regression analysis was used for univariate and multivariate analyses to identify clinical variables related to treatment efficacy. This study included a total of 176 patients who received a combination of lenvatinib and PD-1 inhibitors. Of these, 103 patients received camrelizumab, 44 received tislelizumab, 20 received sintilimab, and 9 received pembrolizumab. There was no significant difference in the pairwise comparison of camrelizumab, tislelizumab, sintilimab, and pembrolizumab using Kaplan–Meier survival analysis. Adverse events occurred in 40 (22.7%) patients (grade ≥ 3, 2.3%). The incidence of grade 3 adverse events among the four PD-1 inhibitor groups was below 5%. Camrelizumab, tislelizumab, sintilimab, and pembrolizumab are viable options for patients with unresectable PLC. These PD-1 inhibitors in combination with lenvatinib showed good safety profiles. The results guide selecting treatment for patients with unresectable PLC.

**Supplementary Information:**

The online version contains supplementary material available at 10.1007/s12672-023-00708-0.

## Introduction

Primary liver cancer (PLC) is a global health concern. It was the sixth most frequently diagnosed cancer and the third leading cause of cancer-related deaths worldwide in 2020, with approximately 906 000 new cases and 830 000 deaths [1]. Liver cancer is associated with a 5-year survival rate of just 18%, making it one of the deadliest cancers. Patients from Asian countries, such as China, have worse outcomes, with 5-year survival rates as low as 12% [2]. Early-stage liver cancers may be cured with surgical resection, ablation, or liver transplantation. Unfortunately, more than 70% of patients with liver cancer are in the middle and late disease stages when they are first diagnosed [3, 4]. Patients with advanced disease lose the opportunity for surgery; such patients can only receive palliative treatment, such as systematic treatment, and their prognosis is poor [5].

In 2007, the FDA approved the multi-targeted tyrosine kinase inhibitor sorafenib, which greatly changed the treatment landscape of hepatocellular carcinoma (HCC) [6]. Lenvatinib is another multi-targeted tyrosine kinase inhibitor (TKI) that can be taken orally and is as effective as sorafenib in inhibiting tumor angiogenesis and growth. As a result, lenvatinib therapy has been included as the second recommended first-line targeted molecular therapy in the 2019 National Comprehensive Cancer Network (NCCN) guidelines [7]. Several successful programs have been completed, leading to the regulatory approval of cabozantinib and ramucirumab, as well as the breakthrough combination therapy of atezolizumab and bevacizumab [8–10]. Despite the promising preliminary results of anti-angiogenic agent and immune checkpoint inhibitor (ICI) combination therapy in advanced HCC, it still encounters various challenges, including the absence of reliable biomarkers to determine treatment response [11–16].

Immune checkpoint inhibitors have been evaluated in clinical trials for the treatment of HCC, but results from single-agent ICI trials have been disappointing. However, immune-based combination therapies have shown more promising results. The combination of atezolizumab and bevacizumab marks a significant advancement in the treatment of HCC and represents a new milestone in the field of HCC treatment [10]. The phase III LEAP-002 study, which compared the combination of lenvatinib and pembrolizumab with the combination of lenvatinib and placebo as first-line treatment for advanced unresectable hepatocellular carcinoma, did not meet the primary endpoints of overall survival (OS) and progression-free survival (PFS) [17]. A result from a large-scale real-world study showed that there was no significant difference in OS between the combination of lenvatinib and pembrolizumab and the combination of lenvatinib with other programmed cell death protein 1 (PD-1) inhibitors [18].

Several studies on the combination of lenvatinib and ICIs have been conducted, and preliminary results show that these combinations are well tolerated [19–22]. Lenvatinib combined with ICIs has shown good antitumor efficacy and safety in patients with liver cancer. Previous studies have explored the efficacy and safety of ICIs combined with lenvatinib and reported their efficacy and adverse event (AE) rates [19, 21, 23–25]. Many clinical trials are still ongoing to investigate the effectiveness of combining lenvatinib with various ICIs [26]. There are significant differences in the drugs used, patients involved, study designs, and phases of research among these clinical trials, and the clinical outcomes are inconsistent. There is still a lack of clinical trials comparing two different ICIs, so it is still unclear what differences in efficacy and safety exist among different ICIs.

Additionally, PD-1 inhibitors developed by different manufacturers vary in composition and molecular structure. Camrelizumab is a humanized monoclonal antibody that binds to the PD-1 receptor [27, 28]. Tislelizumab is another monoclonal antibody that specifically binds to PD-1 with high affinity and specificity [29]. Sintilimab is a recombinant humanized IgG4 monoclonal antibody that targets PD-1 [30]. Pembrolizumab is a humanized IgG4 monoclonal antibody that targets PD-1 [31]. The pharmacokinetic behavior of anti PD-1 antibodies from different companies and the differences in their affinity for the human neonatal Fc receptor (hFcRn) may result in variations in drug efficacy. Furthermore, different PD-1 inhibitors have distinct mechanisms of action and target inhibition efficiency, which may lead to differences in therapeutic effects [27, 28].

To date, there is currently a lack of research exploring whether there are differences in the efficacy and safety of different ICIs combined with lenvatinib, and which combination regimen can bring better benefits to patients. Hence, we aimed to investigate whether there are differences in the efficacy and safety of various PD-1 inhibitors in combination with lenvatinib for the treatment of unresectable PLC.

## Methods

### Study population and data collection

**Fig. 1:**
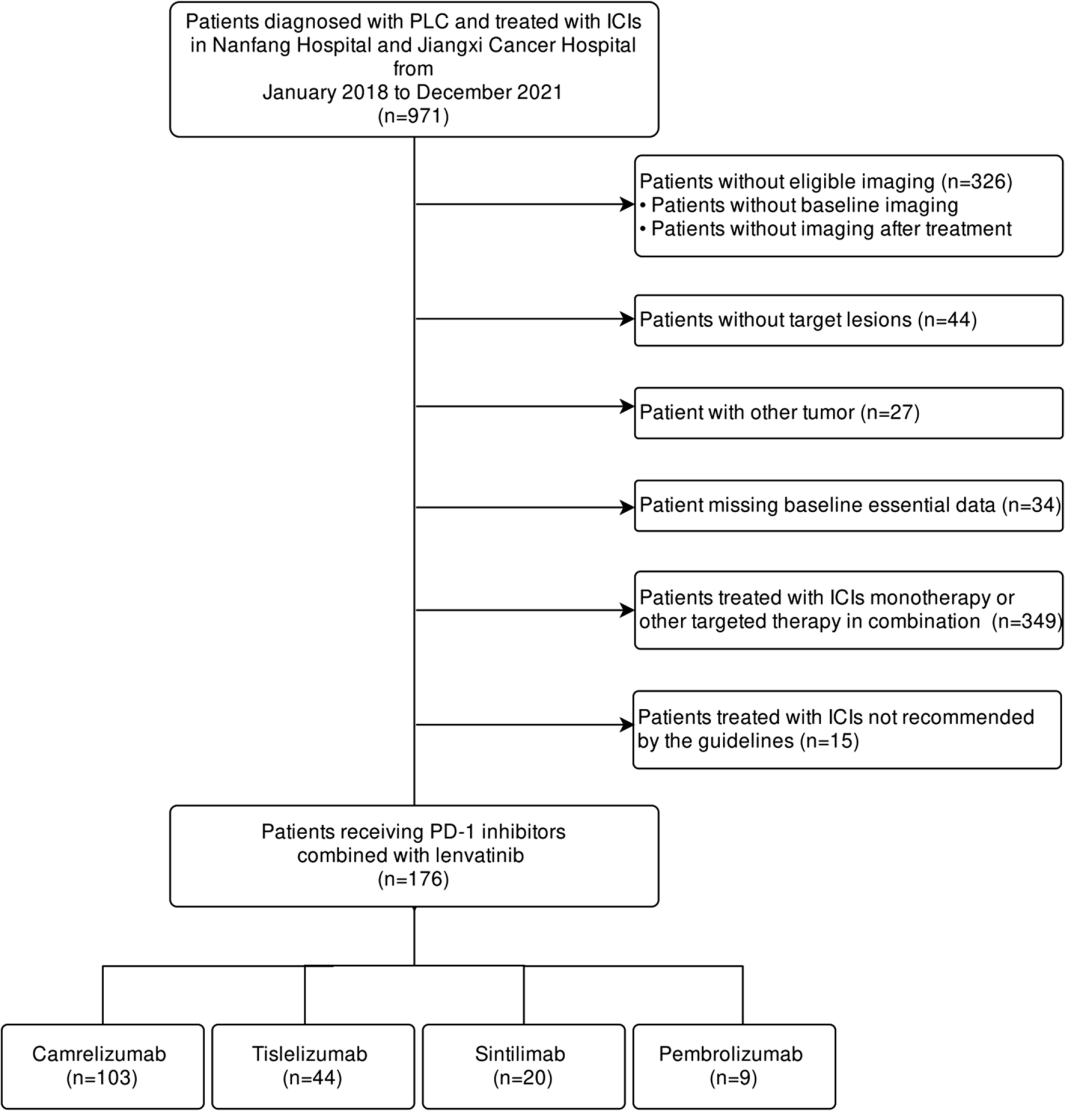
Study flowchart. *PLC* primary liver cancer, *ICI* immune checkpoint inhibitor

Between January 2018 and December 2021, patients who were histologically or clinically diagnosed with PLC according to the criteria of the NCCN Clinical Practice Guidelines [32], and treated with lenvatinib in combination with an ICI at the Southern Hospital and Jiangxi Cancer Hospital, were retrospectively enrolled. The flowchart of the patient selection process is shown in Fig. 1. The inclusion criteria were as follows: (1) patients diagnosed with PLC and treated with ICIs in combination with lenvatinib and (2) patients with Eastern Cooperative Oncology Group performance status (ECOG PS) ≤ 2 points. The exclusion criteria were as follows: (1) patients who had not undergone the required imaging examination before and after treatment; (2) patients without measurable target lesions; (3) patients with tumors other than PLC; (4) patients without baseline data; (5) patients treated with ICI monotherapy; and (6) patients treated with ICIs not recommended by the guidelines. Before treatment with ICIs, the following data of eligible patients were collected: age; sex; cirrhosis presence; hepatitis B virus infection; ECOG PS; Child–Pugh class; Barcelona Clinic Liver Cancer stage (BCLC); portal vein tumor thrombus (PVTT); lines of systemic treatment; type of ICI; tumor metastasis presence; ascites; splenomegaly; routine blood test data including neutrophils and lymphocyte counts, neutrophil to lymphocyte ratio (NLR), hemoglobin (HGB) level; and other laboratory tests. Portal vein tumor thrombus was classified into one of four types [33, 34]. Four PD-1 inhibitors (camrelizumab, tislelizumab, sintilimab, and pembrolizumab) were included in this study.

### Assessment and endpoints

An assessment of treatment response was scheduled at weeks 6–8 using magnetic resonance imaging (MRI) or dynamic computed tomography (CT). Splenomegaly was also diagnosed using CT imaging or MRI. Tumor response, including the objective response rate (ORR) and disease control rate (DCR), was evaluated according to the Response Evaluation Criteria in Solid Tumors, version 1.1 (RECIST 1.1) [35]. The treatment responses were evaluated and classified by a radiologist.

Adverse events and their grades were recorded according to the Common Terminology Criteria for Adverse Events (CTCAE; version 5.0). The primary endpoint was overall survival (OS) and the secondary endpoint was progression-free survival (PFS). Overall survival was defined as the period from the start of PD-1 inhibitor treatment to death. Progression-free survival was defined as the time from the first use of PD-1 inhibitors to disease progression or death.

### Statistical analysis

Descriptive statistics were used to summarise the baseline characteristics, tumor response, and AE data. Kaplan–Meier analysis and log-rank test were used to compare the OS and PFS of patients treated with different PD-1 inhibitors. Cox regression analysis was used for univariate and multivariate analyses to identify clinical variables related to efficacy. All tests were bilateral, and statistical significance was set at *P* < 0.05. In the pairwise comparison of Kaplan–Meier survival analysis, Bonferroni correction was used, and the difference was considered statistically significant when the *P* value was less than 0.0083. SPSS version 26 software (IBM Corp., Chicago, Illinois, USA) and R (version 4.1.0) were used for all statistical analyses.

## Results

### Patient characteristics

One hundred and seventy-six patients who received lenvatinib in combination with PD-1 inhibitors were included in this study. The baseline patient characteristics are summarised in Table 1. Of these, 103 patients received camrelizumab, 44 received tislelizumab, 20 received sintilimab, and 9 received pembrolizumab. Fifty-one patients (29.0%) were aged 60 years or older. One hundred and fifty-three patients (86.9%) were treated with PD-1 inhibitors as the first-line treatment. Most patients had BCLC stage B or C disease, accounting for 19.3% and 80.1%, respectively. One hundred and twenty-seven patients had intrahepatic metastasis, 41 had lung metastasis, and 58 had lymph node metastasis. Eighty-seven patients had extrahepatic metastasis.

**Table 1:** Patient and disease characteristics (N = 176)

Patient characteristic	No	Percent of cohort
Age		
< 60	125	71.0
≥ 60	51	29.0
Sex		
Male	159	90.3
Female	17	9.7
Number of lesions		
Single	25	14.2
Multiple	151	85.8
Number of treatment lines		
1	153	86.9
2	20	11.4
≥ 3	3	1.7
Immunotherapy		
Camrelizumab	103	58.5
Tislelizumab	44	25.0
Sintilimab	20	11.4
Pembrolizumab	9	5.1
ECOG PS		
0	106	60.2
≥ 1	70	39.8
BCLC stage		
A	1	0.6
B	34	19.3
C	141	80.1
Child–Pugh score		
A	132	75.0
B	43	24.4
C	1	0.6
HBV infection		
No	17	9.7
Yes	159	90.3
Antiviral therapy before treatment		
No	34	19.3
Yes	142	80.7
Liver metastasis		
No	49	27.8
Yes	127	72.2
Lung metastasis		
No	135	76.7
Yes	41	23.3
Lymph node metastasis		
No	118	67.0
Yes	58	33.0
Extrahepatic metastasis		
No	89	50.6
Yes	87	49.4
PVTT		
No	86	48.9
Yes	90	51.1
Ascites		
No	123	69.9
Yes	53	30.1
NLR		
< 3	96	54.5
≥ 3	80	45.5
HGB		
≥ 120 g/L	116	65.9
< 120 g/L	60	34.1

*ECOG PS* Eastern Cooperative Oncology Group performance status, *BCLC* Barcelona Clinic Liver Cancer, *HBV* Hepatitis B virus, *PVTT* Portal vein tumour thrombus, *NLR* neutrophil to lymphocyte ratio, *HGB* haemoglobin

### Tumor response and safety

**Fig. 2:**
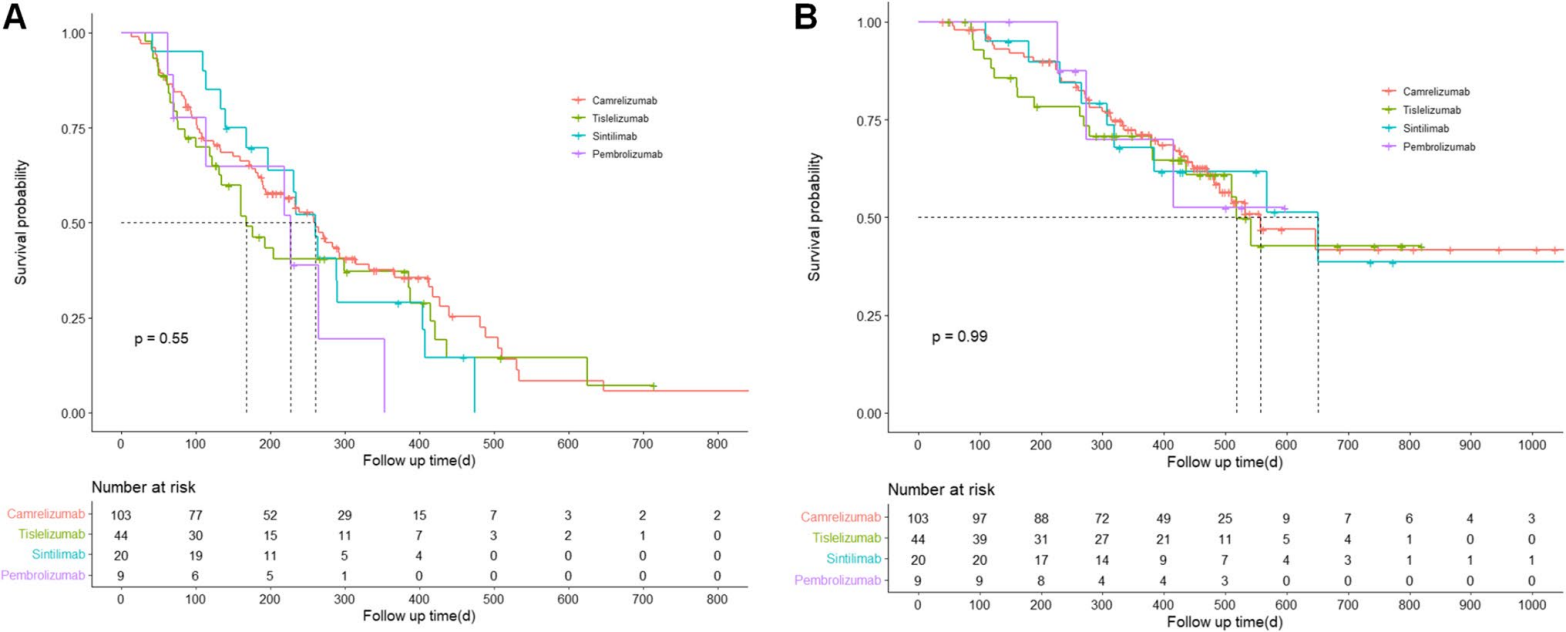
Kaplan–Meier analysis. Progression-free survival (**A**) and overall survival (**B**) in patients receiving different PD-1 inhibitors combined with lenvatinib

The median follow-up duration was 481 days (95% confidence interval (CI): 454–517 days). At the time of data cut-off, 85 patients were still alive and 21 were lost to follow-up. The median OS of all patients was 557 days (95% CI: 439–675 days, mean 658.5 days) and the median PFS was 234 days (95% CI: 186–282 days, mean 286.0 days). The ORR was 20.5% and the DCR was 79.0% for all patients. Thirty-seven patients (21.0%) had progressive diseases as the best response (Table 2). The ORR in the patients treated with camrelizumab, tislelizumab, sintilimab, and pembrolizumab was 23.3%, 15.9%, 20.0%, and 11.1%, respectively. The DCR was 80.6%, 75.0%, 80.0%, and 77.8% in patients receiving camrelizumab, tislelizumab, sintilimab, and pembrolizumab, respectively. The median PFS of the patients receiving camrelizumab, tislelizumab, sintilimab, and pembrolizumab was 260 days (95% CI: 212–308 days), 168 days (95% CI: 113–224 days), 260 days (95% CI: 218–302 days), and 227 days (95% CI: 79–375 days), respectively. The median OS of the patients receiving camrelizumab, tislelizumab, and sintilimab was 557 days (95% CI: 410–704 days), 518 days (95% CI: 470–566 days), and 651 days (95% CI: 312–990 days), respectively. Similar 1-year OS rates were observed among the four groups: 71.2% for camrelizumab, 70.8% for tislelizumab, 67.9% for sintilimab, and 70.0% for pembrolizumab. The Kaplan–Meier survival curves for OS and PFS are shown in Fig. 2. There was no significant difference in the pairwise comparison of patients treated with the different PD-1 inhibitors using Kaplan–Meier survival analysis (*P* > 0.0083) (Table 3). In our study, 156 patients were diagnosed with HCC, 20 with intrahepatic cholangiocarcinoma (ICC), or with HCC-ICC. Another survival analysis was performed on 156 patients diagnosed with HCC. The results of Kaplan–Meier survival analysis and paired comparisons between groups are shown in Fig. S1 and Table S1. No significant difference in the pairwise comparison of patients treated with the different PD-1 inhibitors (P > 0.0083).

**Table 2:** Response outcomes

Outcome	Total (n = 176)	Camrelizumb (n = 103)	Tislelizumab (n = 44)	Sintilimab (n = 20)	Pembrolizumab (n = 9)
Best response					
Partial response	36 (20.5)	24 (23.3)	7 (15.9)	4 (20.0)	1 (11.1)
Stable disease	103 (58.5)	59 (57.3)	26 (59.1)	12 (60.0)	6 (66.7)
Progressive disease	37 (21.0)	20(19.4)	11 (25.0)	4 (20.0)	2 (22.2)
Overall response rate	20.5%	23.3%	15.9%	20.0%	11.1%
Disease control rate	79.0%	80.6%	75.0%	80.0%	77.8%
Median progression-free survival, day	234	260	168	260	227
Median overall survival, day	577	557	518	651	-
1-year overall survival rate	70.7%	71.2%	70.8%	67.9%	70.0%
2-year overall survival rate	41.4%	41.8%	42.7%	38.6%	-

**Table 3:** Pairwise comparison of Kaplan–Meier survival analysis of different PD-1 inhibitors

	Immunotherapy	Camrelizumab		Tislelizumab		Sintilimab		Pembrolizuma b	
		Chi-square	*P*	Chi-square	*P*	Chi-square	*P*	Chi-square	*P*
OS	Camrelizumab			0.111	0.739	< 0.001	0.997	0.001	0.975
	Tislelizumab	0.111	0.739			0.082	0.774	0.086	0.770
	Sintilimab	< 0.001	0.997	0.082	0.774			0.005	0.941
	Pembrolizumab	0.001	0.975	0.086	0.77	0.005	0.941		
PFS	Camrelizumab			0.630	0.427	0.439	0.507	1.667	0.197
	Tislelizumab	0.630	0.427			0.045	0.832	0.387	0.534
	Sintilimab	0.439	0.507	0.045	0.832			1.497	0.221
	Pembrolizumab	1.667	0.197	0.387	0.534	1.497	0.221		

*OS* overall survival, *PFS* progression-free survival

Adverse events occurred in 45 (23.8%) patients (grade ≥ 3, 2.1%). Among them, endocrine AEs were the most frequent (≥ 6.4% of patients) (Table 4). The incidence of AEs in the camrelizumab, tislelizumab, sintilimab, and pembrolizumab groups was 22.3%, 25.0%, 25.0%, and 11.1%, respectively. Grade 3 or higher AEs occurred in patients treated with camrelizumab (2 cases) and tislelizumab (2 cases). One patient who received lenvatinib in combination with tislelizumab stopped PD-1 inhibitor therapy because of grade 4 pneumonia and grade 4 anemia. Two patients who received lenvatinib in combination with camrelizumab stopped immunotherapy owing to adverse cardiac events. No deaths due to fatal AEs were reported.

**Table 4:** Adverse events

	Total (%)	Camrelizumab	Tislelizumab	Sintilimab	Pembrolizumab
Any grade	40 (22.7)	23 (22.3)	11 (25.0)	5 (25.0)	1 (11.1)
Grade ≥ 3	4 (2.3)	2 (1.9)	2 (4.5)	0 (0)	0 (0)
Skin	10 (5.7)	7 (6.8)	2 (4.5)	1 (5.0)	0 (0)
Endocrine	9 (5.1)	5 (4.9)	1 (2.3)	3 (15.0)	0 (0)
Liver	4 (2.3)	1 (1.0)	2 (4.5)	0 (0)	1 (11.1)
Stomach and intestines	4 (2.3)	2 (1.9)	2 (4.5)	0 (0)	0 (0)
Lung	1 (0.6)	0 (0)	1 (2.3)	0 (0)	0 (0)
Kidney	2 (1.1)	2 (1.9)	0 (0)	0 (0)	0 (0)
Blood	7 (4.0)	5 (4.9)	2 (4.5)	0 (0)	0 (0)
Heart	10 (5.7)	4 (3.9)	5 (11.4)	1 (5.0)	0 (0)

### Univariate and multivariate analyses

**Fig. 3:**
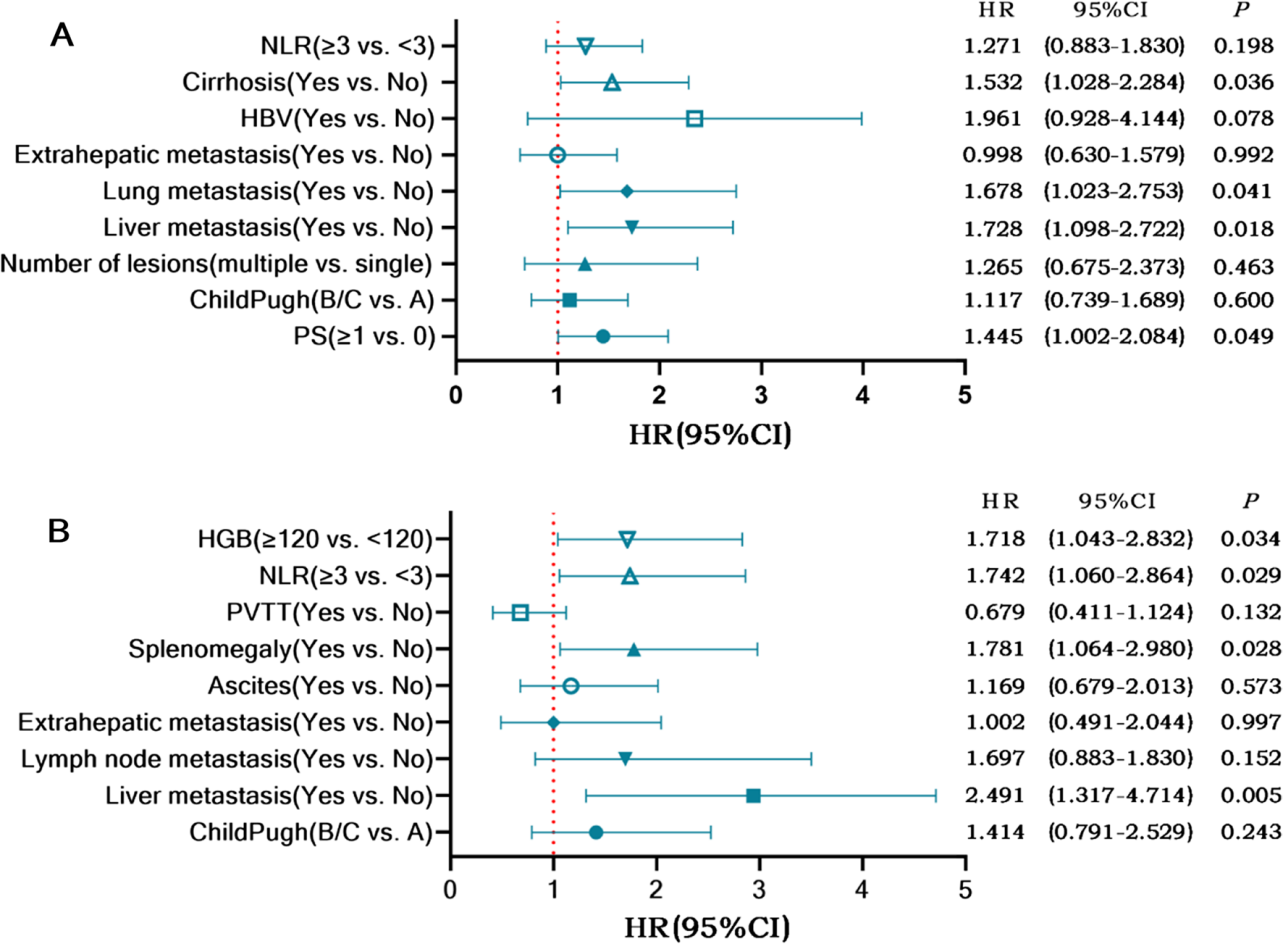
Forest plot of multivariate COX regression analysis for progression-free survival (**A**) and for overall survival (**B**)

In the univariate analysis of PFS, we found that different PD-1 inhibitors were not risk factors for PFS. The multivariate analysis suggested that ECOG PS, cirrhosis, intrahepatic metastasis, and pulmonary metastasis were independent risk factors for PFS (Table S2). The univariate analysis demonstrated that different PD-1 inhibitors were not risk factors for OS. Child–Pugh class, intrahepatic metastasis, lymph node metastasis, extrahepatic metastasis, ascites, splenomegaly, NLR, HGB, and PVTT were included in the multivariate analysis, and the results showed that intrahepatic metastasis, splenomegaly, NLR, and HGB were independent risk factors for OS (Table S3). Forest plots of the multivariate COX regression analysis for PFS and OS are shown in Fig. 3.

## Discussion

In this real-world study, we explored the efficacy and safety of different PD-1 inhibitors in combination with lenvatinib for the treatment of unresectable PLC. There was no significant difference in the efficacy of camrelizumab, tislelizumab, sintilimab, or pembrolizumab combined with lenvatinib. All AEs associated with the PD-1 inhibitors were manageable. Intrahepatic metastasis, splenomegaly, NLR, and HGB were independent risk factors for disease prognosis under treatment with PD-1 inhibitors in combination with lenvatinib.

In clinical trials, the combination of antiangiogenic drugs and ICIs has achieved significant clinical efficacy, with higher ORR and better survival results than monotherapy [10, 19, 36–38]. The combination of ICIs and targeted therapy has good efficacy and safety and will become an indispensable therapeutic option for liver cancer in the future. Future studies should focus on finding a better combination of ICI inhibitors and targeted therapy. Most previous studies have explored the differences between combination therapy and monotherapy, and very few studies have evaluated the difference in efficacy among different ICIs combined with lenvatinib. A study found that there was no significant difference in OS and PFS between using combination of lenvatinib and pembrolizumab versus using combination of lenvatinib and other anti-PD-1. In our study, we further compared the efficacy of four PD-1 inhibitors. A study involving 29 patients showed that lenvatinib combined with nivolumab and lenvatinib combined with pembrolizumab was associated with an ORR of 37.5% and 7.7%, respectively. Kaplan–Meier survival estimates indicated that there was no difference between the groups in terms of OS and PFS [39]. Our study included a larger sample and more types of PD-1 inhibitors, and our results also revealed that there was no significant difference in OS and PFS among various PD-1 inhibitors used in combination with lenvatinib. In addition, the current study included a real-world cohort of patients with unresectable PLC who are typically excluded from prospective clinical trials. Therefore, our results guide the treatment of such patients.

A meta-analysis comparing the efficacy and safety of nivolumab and pembrolizumab in patients with non-small cell lung cancer showed no significant difference in efficacy [40]. Torasawa et al. reported that the efficacy and safety of nivolumab and pembrolizumab in subsequent treatment lines were not significantly different in patients with advanced non-small cell lung cancer [41]. However, patients with PLC were not included in their study. Compared to these studies, our study included a large number of patients with PLC.

In terms of safety, high-grade hepatic toxicity was observed in a previous study, which caused four deaths. However, there was no significant difference in the frequency of AEs between nivolumab and pembrolizumab [39]. Another study showed that the incidence of all-grade AEs was 27%, and the incidence of grade 3 or higher AEs was 6% in patients with advanced hepatocellular carcinoma who received ICI monotherapy [42]. It has been reported that serious AEs after ICI and tyrosine kinase inhibitor combination treatment occur in up to 67% of patients [19]. Although 22.7% of the patients experienced AEs related to PD-1 inhibitors in the current study, all AEs were manageable; there were no fatal AEs, and the four PD-1 inhibitors were well tolerated by the patients. Compared to the aforementioned results, no new or unexpected AEs occurred in our cohort [19, 37, 43].

Our previous study results suggest that lymph node metastasis and splenomegaly is an independent prognostic factor for PLC in immunotherapy [44–46]. A meta-analysis provided strong or highly suggestive evidence that NLR is associated with cancer prognosis [47]. A prospective study showed that a normal pre-treatment HGB level is a favorable prognostic marker in patients with advanced tumors receiving ICI treatment [48]. Our results are similar to those of previous studies.

The present study had some limitations. First, it was a retrospective study that includes a wide range of different patient populations and the study was limited to an Asian population with a relatively small sample size. Owing to the heterogeneity of the study population and the different combination therapies, the results must be interpreted with caution. Second, the number of patients receiving pembrolizumab and sintilimab was relatively small, which reduced the quality of the conclusions. In addition, the median follow-up time of this study was not long enough, and a longer follow-up time is required to obtain more meaningful median OS results in the cohort. Finally, the research results need to be verified in a larger population and prospective studies.

## Conclusions

Our results suggest that camrelizumab, tislelizumab, sintilimab, and pembrolizumab are viable options for patients with unresectable PLC. PD-1 inhibitors combined with lenvatinib have good safety, and this real-world study guides selecting treatment for patients with PLC.

## Supplementary Information

Supplementary file 1 (TIF 14400 KB)

Supplementary file 2 (DOCX 22 KB)

## Data Availability

Data are available from the authors upon reasonable request.

## Abbreviations

AE: Adverse event
BCLC: Barcelona Clinic Liver Cancer
CT: Computed tomography
DCR: Disease control rate
ICI: Immune checkpoint inhibitor
MRI: Magnetic resonance imaging
NCCN: National Comprehensive Cancer Network
ORR: Objective response rate
OS: Overall survival
PFS: Progression-free survival
PLC: Primary liver cancer
PVTT: Portal vein tumor thrombus
