# Supplementary figures and images for: Efficacy and safety of different PD-1 inhibitors in combination with lenvatinib in the treatment of unresectable primary liver cancer: a multicentre retrospective study
Source: Discov Oncol. 2023 Jun 19;14:105. doi: 10.1007/s12672-023-00708-0 (PMC10279630; doi:10.1007/s12672-023-00708-0)

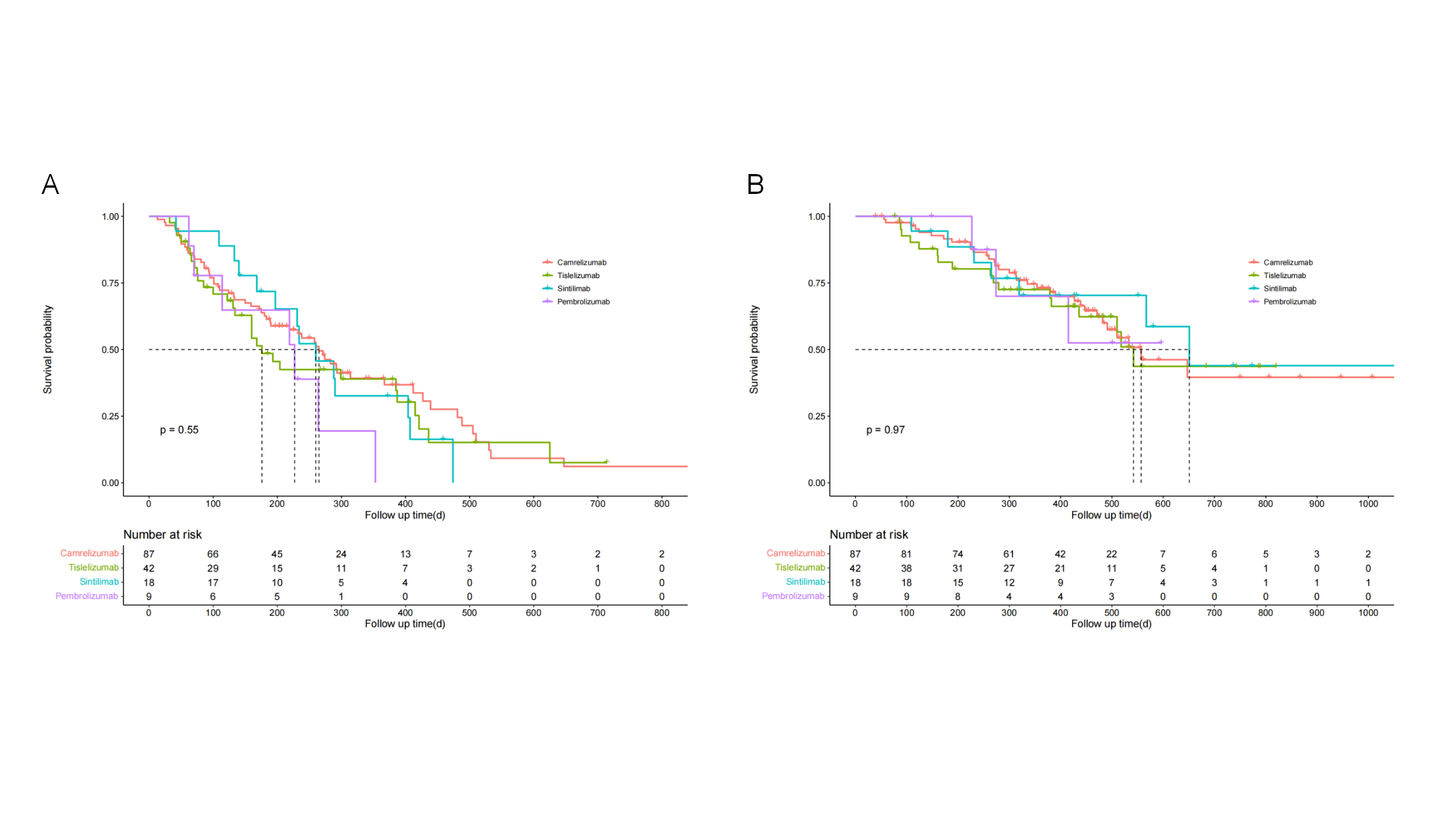

Supplement: Supplementary file 1 — (TIF 14400 KB) [file 12672_2023_708_MOESM1_ESM.tif]
