# Supplementary material for: Efficacy and safety of different PD-1 inhibitors in combination with lenvatinib in the treatment of unresectable primary liver cancer: a multicentre retrospective study
Source: Discov Oncol. 2023 Jun 19;14:105. doi: 10.1007/s12672-023-00708-0 (PMC10279630; doi:10.1007/s12672-023-00708-0)
Supplement: Supplementary file 2 — (DOCX 22 KB) [file 12672_2023_708_MOESM2_ESM.docx]

Table S1. Pairwise comparison of Kaplan–Meier survival analysis of different PD-1 inhibitors in 156 HCC patients.

|  | Immunotherapy | Camrelizumab | | Tislelizumab | | Sintilimab | | Pembrolizumab | |
| --- | --- | --- | --- | --- | --- | --- | --- | --- | --- |
|  |  | Chi-square | *P* | Chi-square | *P* | Chi-square | *P* | Chi-square | *P* |
| OS | Camrelizumab |  |  | 0.111 | 0.739 | ＜0.001 | 0.999 | 0.001 | 0.974 |
|  | Tislelizumab | 0.111 | 0.739 |  |  | 0.082 | 0.774 | 0.086 | 0.770 |
|  | Sintilimab | ＜0.001 | 0.999 | 0.082 | 0.774 |  |  | 0.005 | 0.941 |
|  | Pembrolizumab | 0.001 | 0.974 | 0.086 | 0.770 | 0.005 | 0.941 |  |  |
| PFS | Camrelizumab |  |  | 0.630 | 0.427 | 0.435 | 0.510 | 1.667 | 0.197 |
|  | Tislelizumab | 0.630 | 0.427 |  |  | 0.045 | 0.832 | 0.387 | 0.534 |
|  | Sintilimab | 0.435 | 0.510 | 0.045 | 0.832 |  |  | 1.497 | 0.221 |
|  | Pembrolizumab | 1.667 | 0.197 | 0.387 | 0.534 | 1.497 | 0.221 |  |  |

Table S2. Univariate and multivariate cox multivariate analyses of progression-free survival.

| Variable | Univariate analysis | | | Multivariate analysis | | |
| --- | --- | --- | --- | --- | --- | --- |
|  | HR | 95% CI | P | HR | 95% CI | P |
| Age（≥60 vs. <60 years） | 1.127 | (0.768–1.653) | 0.541 |  |  |  |
| Sex (female vs. male) | 1.072 | (0.574–2.002) | 0.826 |  |  |  |
| Number of lesions (multiple vs. single) | 1.873 | (1.051–3.338) | 0.033 | 1.265 | (0.675–2.373) | 0.463 |
| Immunotherapy |  |  | 0.552 |  |  |  |
| Tislelizumab vs. Camrelizumab | 1.189 | (0.777–1.817) | 0.425 |  |  |  |
| Sintilimab vs. Camrelizumab | 1.172 | (0.678–2.026) | 0.57 |  |  |  |
| Pembrolizumab vs. Camrelizumab | 1.68 | (0.767–3.680) | 0.195 |  |  |  |
| ECOG PS (≥1 vs. 0) | 1.447 | (1.010–2.037) | 0.044 | 1.445 | (1.002–2.084) | 0.049 |
| Child–Pugh score (B/C vs. A) | 1.236 | (0.831–1.838) | 0.296 | 1.117 | (0.739–1.689) | 0.6 |
| HBV (yes vs. no) | 2.451 | (1.195–5.029) | 0.014 | 1.961 | (0.928–4.144) | 0.078 |
| Cirrhosis (yes vs. no) | 1.64 | (1.120–2.402) | 0.011 | 1.532 | (1.028–2.284) | 0.036 |
| Liver metastasis (yes vs. no) | 1.861 | (1.227–2.824) | 0.003 | 1.728 | (1.098–2.722) | 0.018 |
| Lung metastasis (yes vs. no) | 1.726 | (1.160–2.567) | 0.007 | 1.754 | (1.068–2.880) | 0.041 |
| Extrahepatic metastasis (yes vs. no) | 1.439 | (1.005–2.060) | 0.047 | 1.009 | (0.644–1.580) | 0.97 |
| NLR (≥3 vs. <3) | 1.449 | (1.016–2.066) | 0.041 | 1.322 | (0.914–1.911) | 0.138 |

ECOG PS: Eastern Cooperative Oncology Group performance status; BCLC: Barcelona Clinic Liver Cancer; HBV: hepatitis B virus; PVTT: portal vein tumour thrombus; NLR: neutrophil to lymphocyte ratio.

Table S3.Univariate and multivariate cox multivariate analysis of overall survival.

| Variable | Univariate analysis | | | Multivariate analysis | | |
| --- | --- | --- | --- | --- | --- | --- |
|  | HR | 95% CI | P | HR | 95% CI | P |
| Age (≥60 vs. <60 years) | 1.396 | (0.856–2.276) | 0.182 |  |  |  |
| Sex (female vs. male) | 1.12 | (0.510–2.458) | 0.777 |  |  |  |
| Immunotherapy |  |  | 0.988 |  |  |  |
| Tislelizumab vs. Camrelizumab | 1.101 | (0.631–1.922) | 0.734 |  |  |  |
| Sintilimab vs. Camrelizumab | 1.004 | (0.485–2.078) | 0.991 |  |  |  |
| Pembrolizumab vs. Camrelizumab | 0.966 | (0.299–3.128) | 0.955 |  |  |  |
| ECOG PS (≥1 vs. 0) | 1.465 | (0.915–2.345) | 0.11 |  |  |  |
| Child–Pugh score (B/C vs. A) | 2.174 | (1.329–3.556) | 0.002 | 1.414 | (0.791–2.529) | 0.243 |
| Liver metastasis (yes vs. no) | 2.695 | (1.443–5.033) | 0.002 | 2.491 | (1.317–4.714) | 0.005 |
| Lymph node metastasis (yes vs. no) | 1.682 | (1.047–2.702) | 0.032 | 1.697 | (0.823–3.503) | 0.152 |
| Extrahepatic metastasis (yes vs. no) | 1.689 | (1.046–2.728) | 0.032 | 1.002 | (0.491–2.044) | 0.997 |
| Ascites (yes vs. no) | 2.31 | (1.381–3.864) | 0.001 | 1.169 | (0.679–2.013) | 0.573 |
| Splenomegaly (yes vs. no) | 2.24 | (1.397–3.593) | 0.001 | 1.781 | (1.064–2.980) | 0.028 |
| PVTT (yes vs. no) | 0.907 | (0.568–1.450) | 0.684 | 0.679 | (0.411–1.124) | 0.132 |
| NLR (≥3 vs. <3) | 2.037 | (1.268–3.275) | 0.003 | 1.742 | (1.060–2.864) | 0.029 |
| HGB (<120 vs. ≥120 g/L) | 2.261 | (1.408–3.630) | 0.001 | 1.718 | (1.043–2.832) | 0.034 |

ECOG PS: Eastern Cooperative Oncology Group performance status; BCLC: Barcelona Clinic Liver Cancer; HBV: hepatitis B virus; PVTT: portal vein tumour thrombus; NLR: neutrophil to lymphocyte ratio; HGB: haemoglobin.
